# Recruitment Issues in Emerging Adult Populations: Focus on Adult Congenital Heart Disease

**DOI:** 10.3390/nursrep10020017

**Published:** 2020-12-02

**Authors:** Laura Hays, Jean McSweeney, Anita Mitchell, Christina Bricker, Angela Green, Reid D. Landes

**Affiliations:** 1 College of Nursing, University of Arkansas for Medical Sciences, Little Rock, AR 72205, USA; mcsweeneyjeanc@uams.edu (J.M.); amitchell@uams.edu (A.M.); 2 College of Nursing, University of South Florida, Tampa, FL 33620, USA; cbricker@usf.edu; 3 Johns Hopkins All Children’s Hospital, St. Petersburg, FL 33701, USA; angela.green@jhmi.edu; 4 Department of Biostatistics, University of Arkansas for Medical Sciences, Little Rock, AR 72205, USA; rdlandes@uams.edu

**Keywords:** recruitment issues, emerging adults, ACHD, congenital heart disease, young adults, recruitment strategies, research recruitment

## Abstract

High-quality nursing research is important to healthcare and is precipitated by successful participant recruitment. Young adults aged 18 to 30 years are particularly difficult to recruit due to transitions during this time, which makes it more problematic to locate these individuals and may make it more difficult for them to prioritize the need for participation. This paper includes data from two cross-sectional survey design pilot studies that aimed to enroll young adults with congenital heart disease using a variety of recruitment methods. The number of participants enrolled in these two pilot studies (7 and 22) was much lower than expected but the recruitment challenges encountered were consistent with other research studies that have recruited young adult populations. After presenting these data and a discussion of the relevant literature, we conclude with proposed strategies for research recruitment of young adults for nurse scientists who directly impact evidence-based literature and practice with research contributions.

## 1. Introduction

The most commonly occurring challenge in research studies is reaching targeted populations. Research recruitment delays result in study extensions and/or increased costs from avoidable protocol amendments. These delays, therefore, also extend the delivery of needed healthcare treatments and interventions to populations who may potentially benefit from the research [1]. Young adults are particularly difficult to successfully enroll in research studies [2]. The purpose of this manuscript is a discussion of the methods employed to reach young adults, including recruitment issues and proposed strategies that may contribute to greater success in this endeavor for future studies.

Recognition of common recruitment issues was a driving factor in the launch of the *All of Us* Research Program, a key piece of the National Institutes of Health’s Precision Medicine Initiative [3]. Individuals aged 18 to 30 years transition to self-sufficiency during this time period and may make frequent changes in living, academic, and work status, making it difficult to locate potential research participants. They may not be willing to participate in or even be knowledgeable about initiatives such as the *All of Us* campaign. This recognized instability and lack of knowledge about research in young adulthood contribute to the complexity of developing and implementing a successful recruitment plan for those within this age group. A lack of normality associated with an extended period of development defined as neither adolescence nor adulthood, usually between the ages of 18 and 25 years, during which an individual closely examines opportunities available at no other point over the life course, has been coined “emerging adulthood” and is the foundation of Arnett’s Theory of Emerging Adulthood [4]. Arnett’s theory tells us that role exploration and identity seeking take high priority during this developmental level and may result in wide variability across this biological age group due to differing subjective psychosocial maturity levels [4]. This variability may contribute to difficulty when recruiting with homogeneous methods.

Keegan and Parsons reviewed studies examining clinical trial enrollment of adolescent and young adult (AYA) cancer patients aged 15 to 39 years published since 2010 and reported significantly lower clinical trial participation for AYAs compared to other age groups [2]. They cite a time of intense change and development, which contributes to decreased research participation and awareness of the importance of the role of research [2]. The alarming result of this lower enrollment in clinical trials for AYAs with cancer has contributed to stagnation in mortality rates compared to other age groups that have greater participation in clinical trials. In response to this survival deficit, the National Cancer Institute Progress Review Group made recommendations for increasing clinical trial enrollment in the AYA population. These recommendations included collaborations to increase community practice setting recruitment, national-level mechanisms to build data banks, and contemporary strategies such as social media to raise awareness of clinical trials [5].

Young adults with congenital heart disease (YACHD) is one such population that is also unique because of its genesis in recent years. Congenital heart disease (CHD) is the presence of a defect in the structure of the heart at birth and is the most common birth defect, present in approximately one of 100 live births [6,7]. In recent decades, advanced surgical repairs for these defects have produced a new population of substantial size: YACHD. As this emerging population’s expected life span has now exceeded the lifespan of previous populations with CHD, their immediate health and long-term self-management needs are unknown. These YACHD must also transition to adulthood, just as those without CHD, and have frequent changes in academic status and living arrangements, like other young adults. Therefore, accessing this population for research studies has similar challenges as the general young adult population.

### 1.1. Recruitment in the Young Adult Population

Difficulty with recruitment in young adult populations is not a new phenomenon. The first wave of the St. Louis Epidemiologic Catchment Area (ECA) project in 1981–1982 (*N* = 3437) investigated sociodemographic characteristics to see which were determinants of difficulty in research recruitment. The results showed that being a young adult was positively associated with increased contact efforts in recruitment, independent of other variables [8].

In more recent years, Nolte, Shauver, and Chung conducted a pilot study (*N* = 374) with adults aged 18 years and older to test four methods of recruitment to gather normative data for the Michigan Hand Outcomes Questionnaire [9]. They used flyers, email, Facebook, and an institution-specific clinical research recruitment website to reach participants. Their study data indicated that Facebook was the most effective method for the recruitment of young adults aged 18 to 37 years, followed by a clinical research recruitment website. In contrast, email was most effective for older adults aged 68 to 77 years. Recruitment via the institution-specific clinical research recruitment website resulted in a study sample that was most age-distributed to match the entire US population [9]. Young, Ballard, and Cooper reported success using community-based recruitment strategies, such as neighborhood cookouts, flyer distribution, and street outreach, for an online survey to investigate opioid use in young adults aged 18 to 35 years in rural areas of Kentucky [10]. The paired face-to-face and web-based recruitment strategy offered a personalization the research team deemed critical in a rural setting and resulted in the procurement of 88 percent of their final sample [10]. Table 1 summarizes recent literature reviews that further discuss research recruitment when sampling young adult populations.

**Table 1:** Summary of literature with discussion of research recruitment when sampling young adult populations.

First Author, Year, Study Design	Included Studies	Recruitment Methods in Included Studies	Recruitment Results in Included Studies
Fern, 2017 Exploratory review	13 studies; 8 discussion papers; 2 reflection papers on recruitment in specific studies; 1 review of National Institutes of Health (NIH) National Cancer Institute initiatives to increase adolescent and young adult participation in cancer clinical trials; 1 secondary analysis	Most studies described used retrospective databases	Interventions recommended: - Early engagement and patient information - Increase research studies in this population - Increase public awareness and provider education about research - Expand and improve recruitment accessibility - Consider amending age eligibility criteria
Hudson, 2017 Systematic review	215 studies; 152 qualitative; 54 quantitative; 9 mixed methods; Studies included children and young adults ages 0–25 years with life-threatening illnesses	17% by letter 10% in person 1% by email 1% by telephone 4% other 68% not reported	49 studies provided information for recruitment rates - 31% of those recruited <50% of eligible participants
Reagan, 2019 Integrative review	18 studies which used Facebook (FB) and at least one other method for recruitment; >80% of participants aged 18 years and above	Paid FB ads Addition of unpaid FB and Craigslist posts in last 6 months of recruitment In person/”traditional” recruitment	- 19.2% participant enrollment rate - 44% participants obtained when used - Approximately 50% of recruitment obtained when used

Fern, 2017 [11]; Hudson, 2017 [12]; Reagan, 2019 [13].

### 1.2. Barriers to Recruitment

Countries with centralized medical registries have the ability to collect and maintain data through electronic health records. For example, the Scottish Health Research Register (SHARE) is a recruitment database that is linked to the National Health Service’s data sets through unique patient identifiers used in the Scottish health service. SHARE can be used to identify research studies that may be appropriate for those who have registered. They report that over 130,000 persons have registered since its inception in 2011 [14]. Unfortunately, the US lacks an organized national database to collect data on long-term outcomes correlated with CHD repairs, making identification and recruitment of a national sample of YACHD very difficult.

Recruitment for YACHD is further complicated by the special health care delivery for this population in the US, which is divided between pediatric and adult facilities. Since 2010, the adult CHD population has surpassed the pediatric CHD numerically [15]; however, many adults continue to be treated in pediatric facilities due to the number of highly specialized providers and nurses who are trained in the unique cardiac anatomies of CHD. Organizations such as the Children’s Heart Foundation and the Adult Congenital Heart Association award grants and fellowships for the advancement of adult CHD research. Thus, researchers who seek to recruit YACHD for research studies must reach out to both adult and pediatric facilities and organizations to locate potential participants, making recruitment more complex than being able to recruit from single sites.

## 2. Materials and Methods/Results

This paper is based on our own research experiences and is supported by the two pilot studies described below. The studies were conducted in accordance with the Declaration of Helsinki, and the University of Arkansas for Medical Sciences Institutional Review Board (IRB) provided ethical approval (Study 1: Protocol 205276; Study 2: Protocol 207273).

### 2.1. State and Correlation of Knowledge and Self-Efficacy of Adults with Congenital Heart Disease in Arkansas: A Pilot Study (Study 1)

The purpose of this pilot study, conducted in 2016, was to determine YACHD’s level of knowledge about potential long-term disease complications and to determine whether there was a relationship between this knowledge and self-efficacy. To be eligible, participants had to reside in Arkansas and had to have received their initial surgical repair at Arkansas Children’s Hospital. This hospital serves the entire state and surrounding areas and is affiliated with the University of Arkansas for Medical Sciences. The University of Arkansas for Medical Sciences providers, practicing on the Arkansas Children’s Hospital campus, see patients with CHD throughout their lifespans. Inclusion criteria included self-report of initial heart defect repair under the age of 12 months in order to ensure they had lived with the life experiences of CHD through infancy, childhood, and adolescence.

We used a correlational, cross-sectional mixed-methods survey study design. We mailed questionnaires to 300 YACHD subjects aged 18 to 25 years in Arkansas who were in the adult CHD database of the Adult Congenital Heart Disease Program of the University of Arkansas for Medical Sciences at Arkansas Children’s Hospital. This database contains names and demographics of the over 1800 adults with CHD who have been treated and are followed at Arkansas Children’s Hospital. Three-hundred subjects aged 18 to 30 were randomly selected from the database to receive mailed study invitations and a research packet. The research packet included (1) the Leuven Knowledge Questionnaire for Congenital Heart Disease, (2) the Stanford Chronic Disease Self-Efficacy Scales, (3) a demographic data sheet for additional information, and (4) four additional open-ended questions to capture any information the participants deemed important about self-management that was not addressed in the other questionnaires [16,17]. Completion of the questionnaires and demographics sheet was expected to take 30 min and implied consent.

#### Study 1 Results

The US Postal Service returned 73 of the 300 packets as “unable to forward.” We searched websites such as www.voterrecord.org and www.whitepages.com to obtain updated addresses for these persons’ returned questionnaires, resulting in 35 of the 73 returned mail-outs being sent to an updated address. Only two of these updated packets were returned undelivered. Initially, 12 potential participants returned completed questionnaire sets. Six were excluded for not meeting inclusion/exclusion criteria (no surgery as an infant or caregiver completed). Six of these respondents met the inclusion criteria, and we retained their completed questionnaires for the study. One additional participant responded because of the follow-up mail-out survey, making a total of 7 qualified participants.

Based on these results, we learned that many addresses of potential participants were not valid from the high number of study packets that were returned undelivered. Additionally, we did not know if the study packets that were delivered with no response reached the addressees, as young adults may be in college or living on their own and not receive mail forwarded from parents or previous households. Based on what we learned from Study 1 and recommendations from the literature [9], we revised our recruitment strategy for Study 2.

### 2.2. Self-Reported Self-Management of Adults with Congenital Heart Disease (Study 2)

Based on both the known and unknown factors discussed previously in what we learned from Study 1, we totally modified our recruitment strategies for Study 2 and sought a national study sample through online survey methods. We conducted a descriptive mixed-methods study in 2017–2018 to gain an understanding and knowledge of what YACHD perceived as important for self-management and to describe those needs across demographic factors, developmental characteristics, cardiac lesion severity, and perceived quality of life. The findings from this study are published elsewhere [18].

Recruitment targeted YACHD that were 18 to 30 years of age who had initial defect repair under the age of 12 months. No power analysis was performed as there were no hypotheses to test. Alternatively, sample precision, as measured by the margin of error for 95 percent confidence intervals for a proportion, was considered in determining adequate sample size [19]. The recruitment goal for this study was 100 to 200 participants, which meant that the results from this study would have a margin of error no larger than 10 to 6.5 percentage points.

Since we knew that this age group responds better to technology-based recruitment [9], we switched from mailing research packets to an online format and used SurveyMonkey. The SurveyMonkey link consisted of four tools: (1) the investigator-developed ACHD Self-Management Experience Questionnaire, (2) the investigator-developed ACHD Demographic Questionnaire, (3) the Adaptive Behavior Assessment System, 3rd Edition, and (4) the Stanford Patient Education Research Center’s Quality of Life Visual Numeric [20]. Completion of the online survey was expected to take 30 min, and completion indicated consent.

#### 2.2.1. Study 2 Recruitment

Because mail-out methods used in Study 1 produced such a low sample size, other recruitment methods with national reach were employed in Study 2. The national Adult Congenital Heart Association (ACHA) supported initial recruitment for this study by posting the information on their website. The ACHA is an organization founded in 1998 and dedicated to improving and extending the lives of those born with heart defects through education, advocacy, and the promotion of research. The ACHA reports more than 11,000 registered members of its discussion forums. The Member Outreach Manager estimated approximately 20 percent of this cohort to be between 18 and 30 years of age. The Outreach Manager posted a description of the purpose of the study and what was required to participate on the website www.achaheart.com. This information supplied the email address for the Primary Investigator (PI), for those interested in participating, in order to request the survey link. Qualifying participants had the option to provide their mailing addresses after completion of the surveys to receive a $20 Amazon gift card to reimburse their time.

#### 2.2.2. Study 2 Results

After 2.5 months of recruitment through the ACHA, only 16 participants had responded. Therefore, we developed alternative recruitment strategies that included recruitment through local, regional, and national hospital- and clinic-based support groups, social media, and snowball sampling. After the IRB approved this modification, we employed these methods and posted the information sheet and study link on sites of major adult-CHD support groups, including the Wisconsin Adult Congenital Heart Support Group, the Adult CHD Survivors of Cincinnati, and the Adolescent and Adult Congenital Heart Disease Online Community of Nationwide Children’s Hospital. In addition, we posted it on the discussion post site of the CHD Adult Support Group of the American Heart Association (AHA). We then sent emails to 65 members of the Society of Pediatric Cardiovascular Nurses (SPCN), with the study information sheet and study link, eliciting help with recruitment through their adult CHD clinics. The membership of the SPCN includes nurses and providers involved in the care of those with CHD. After four additional months of recruitment, we collected 9 additional study responses, making it a total of 25 study responses. On examination of the responses to evaluate eligibility, 22 of the responses met eligibility criteria and were retained for analysis.

We next sought volunteer sites. Mended Little Hearts is a volunteer support network for individuals and families affected by congenital heart disease. This organization began in 2004 as an extension of Mended Hearts, which is the largest peer-to-peer heart patient support network in the world for all ages. Together, the two groups have 300 chapters and are active in more than 460 hospitals in North America. We submitted a request to their national approval committee for permission to post the study information and SurveyMonkey link on their closed Facebook pages. Unfortunately, our request was denied because Mended Little Hearts had several other research studies already sponsored on their sites at that time.

After two additional months with no further participants, we submitted and obtained IRB approval to place advertisements in online and print newspapers in large cities where there are pediatric hospitals known for managing large populations of YACHD. We placed these weekend advertisements in online and print newspapers in the Cincinnati Enquirer (Cincinnati and Kentucky areas), San Francisco Chronicle, Los Angeles Times (Orange County), and the New York Times Nationwide edition, which is heavily distributed in the northeastern US. After one month, no additional participants responded to recruitment from these newspaper advertisements.

We closed recruitment with 22 participants after 9.5 months, well short of our original goal of 100 to 200 participants. A sample size of 22 participants would result in a 95% margin of error of 21 percentage points, maximum.

## 3. Discussion

Effective recruitment is imperative to provide evidence-based research. Without participants to inform research studies, we cannot advance scientific methods to improve the lives and health of populations worldwide. Online and social media recruitment methods are efficient, low-cost means of reaching large numbers of participants compared with traditional methods [21,22,23]. Some research has reported these methods were successful for the recruitment of young adult populations [24,25]. In contrast, we and others have struggled with research recruitment among young adults, even when using the methods that are reportedly most acceptable to young adults [26].

Demographically representative national databases and research participant registries are key to providing a readily available sample for research studies [27,28,29]. Young adults may not perceive the need to seek out a research study or to consider participation a priority with the other developmental tasks they are navigating during emerging adulthood. A registry facilitates contact among researchers and individuals who have shown an interest and willingness for participation and, therefore, can effectively shorten the recruitment process. Bonner, Cragun, Reynolds, Vadaparampil, and Pal used the Florida cancer registry for a clinical research study investigating recruitment methods for young Black women with breast cancer [30]. They found that challenges with recruitment of this underserved young adult population included higher uninsured rates and lower healthcare utilization compared to non-Hispanic whites. Common sampling methods are used to access potential participants from large academic centers or healthcare systems, which may miss those individuals. Bonner et al. found that recruitment using the state cancer registry was a feasible method of reaching this otherwise underrepresented population with demographic variables representative of the area [30].

Researchers in the state of Arkansas launched a research participant registry in 2016, which now houses over 6900 willing potential research participants who are representative of 2018 Arkansas demographics [31]. Registrants select from over 25 health interest categories upon registration, including “healthy volunteer”. Registries like this one increase the validity of studies with the generalization of results, decrease costs by eliminating delays in recruitment, and shorten the time to dissemination.

Research has shown that recruitment from clinic-based sampling frames are not always representative of their surrounding communities and may have limited feasibility due to their finite cohorts of potential participants, which expand at rates less than may be required to meet recruitment goals for large-scale research studies [32,33]. Oakes et al. compared a sample gleaned from a uniform electronic health record system of an affiliated healthcare system in a metropolitan area to demographic characteristics from the 2010 US Decennial Census and the Census Bureau’s American Community Survey pooled from 2006 to 2010 (*N* = 14,321) [32]. They reported that individuals in the clinic’s database were more educated compared to the geographic area represented (42 versus 34 percent with a bachelor’s degree or higher, respectively), although individual-level data differed on both race and education and so it is unclear whether the educational difference reported was due to self-reported biases within the sampling frame.

The *All of Us* Research Program began collecting health data and biospecimens in 2018 of individuals who reflect the diversity of the United States, with a goal of having one million or more participants within five to six years. The research portal for access to query the data opened in 2020 for approved users [3]. During this initial use phase, select researchers help to finetune processes that continue to evolve the capabilities of the growing dataset. Programs like this one will assist in addressing the challenges of obtaining diverse generalizable samples to provide the best evidence-based state of scientific research. It will provide a cost-effective means to recruit and should substantially decrease delays. It is important that nursing science incorporates and develops other novel recruitment strategies, such as this one and others, to support nursing research and build evidence-based nursing literature.

A major recruitment challenge encountered with the YACHD population could be related to their inordinately high lost-to-follow-up status. The AHA estimates that appropriate specialized providers see fewer than 30 percent of CHD adults in the US for follow-up [34,35]. Significant care gaps with transitioning ACHD exist globally as well; researchers in the United Kingdom reported that 24 percent of a large cohort study of patients with repair of Tetralogy of Fallot (*N* = 1085) were not currently registered with specialized cardiology clinics [36]. Mackie et al. also found that 61 percent of ACHD aged 18 to 22 in Quebec, Canada (*N* = 643), had not received cardiac follow-up care [37]. As a result, current contact information is unknown for many YACHD, and successful research study recruitment of these young adults is dependent on choosing appropriate, targeted recruitment methods.

Although a high lost-to-follow-up ratio may offer insight into recruitment difficulties with the emerging YACHD population, there are still undefined barriers to research recruitment in young adult populations in general [38,39]. Partridge et al. described strategies used to recruit 18- to 35-year-old participants into a randomized controlled trial to prevent weight gain (*N* = 250) [26]. As we did with our study, they adjusted their original recruitment goal of 354 participants after experiencing recruitment difficulties. The first phase of their recruitment invited participation via letters of invitation from patients’ general practitioners, which provided instruction and the link to an online survey for eligibility screening. These letters of invitation from practitioners resulted in 68 participants. The second phase of their recruitment included print media (such as brochure distribution and poster placement), which resulted in 105 participants; electronic media (such as Facebook, Google, and university e-newsletters), which resulted in 68 participants; snowballing, which resulted in five participants. This decrease in the sample size resulted in their statistical inability to achieve 80 percent power [26].

Another study investigated strategies to reach young adults aged 18 to 35 years in North Carolina and Rhode Island (*N* = 599) [40]. This study was part of SNAP, a multicenter randomized trial to compare weight gain prevention approaches. Researchers employed multiple recruitment strategies, including community events, internet advertisements, mass emails, and newspaper advertisements; however, direct mail was the most successful recruitment method reported. As with Partridge’s study [26], potential participants for Crane’s study were directed online to begin their participation [40]. Even so, Crane et al. only reported a 0.9 percent response rate for mail-out recruitment, measured by the number of study screens initiated [40].

A shared recruitment strategy in Partridge et al.’s and Crane et al.’s studies that was successful was the combination of a paper product (such as a letter of invitation, direct mail, or other print media) and a link to a survey or website for the potential respondents to continue participation after reviewing [26,40]. Lam, Partridge, and Allman-Farinelli conducted a systematic review to examine strategies employed to recruit young adults aged 18 to 35 [41]. Twenty-six studies published between 1998 and 2014 were included. Results revealed that mixed recruitment strategies yielded the greatest research study participants, with a passive recruitment method followed by an active recruitment method most successful.

Our Study 1 used a mail-out strategy with stamped envelopes for returning surveys with poor results. Study 2 used a purely online recruitment strategy that provided a link to an online survey, and results were still less than optimal. As revealed by the studies discussed here, perhaps a melding of the two methods would yield the greatest recruitment results when targeting emerging adult populations. This paper is limited by the small size of our pilot studies. The inability to achieve targeted recruitment rates in young adult populations is itself a barrier to understanding methods of best practices for recruitment.

### Implications for Nursing

Nursing research plays an integral part in developing evidence-based interventions that aid populations such as ACHD to achieve and maintain optimal health and quality of life. The nursing profession must lead in the development of registries and methods for recruitment of young adults for research studies so that we can know their needs based on a diverse generalized sample.

It is still unclear, ultimately, how best to successfully recruit young adults for research studies. There is a recurrent propensity in the literature for the coupling of a print document and an online follow-up inquiry that results in higher recruitment among young adults. We used a mail-out survey in one study and online recruitment in another study but did not combine the methods. Future research should investigate combinations of print and online recruitment strategies when recruiting young adult populations, such as a printed brochure with directions to a website to login and complete a survey or register for a focus group. Qualitative research studies are needed to explore the reasons for research participation hesitancy from young adults’ perspectives.

Perhaps among the most important roles of nurses is patient education of the importance of research, especially for YACHD. Patients must understand the impact that research has on their lives and the lives of others and on the development of self-management interventions and disease treatments. Understanding the benefit that research provides, both societally and personally, may help to motivate young adults to seek out participation in research studies and increase research recruitment of young adult populations. Table 2 summarizes recommendations for improving future research recruitment outcomes.

## 4. Conclusions

Challenges with recruiting young adults for research studies persist despite evolving sampling strategies. Our experiences are with the YACHD population and mirror that of researchers of other young adult populations. We offer recommendations for future research that build on these experiences.

## Figures and Tables

**Table 2:** Recommendations to improve future research recruitment outcomes.

Use multiplex approaches to population-targeted research recruitment.
Prioritize demographically representative research participant registries to include development and use.
Provide patient education on the importance and impact of research studies to promote participation.
